# Suicide risk communication and intervention preferences for veterans and service members

**DOI:** 10.3389/fpubh.2023.1215925

**Published:** 2023-11-21

**Authors:** Abby E. Beatty, John S. Richardson, Sonja V. Batten, Steven Weintraub, Karen Hogan, Keith Hotle

**Affiliations:** ^1^Stop Soldier Suicide, Durham, NC, United States; ^2^Veteran Tickets Foundation, Tempe, AZ, United States

**Keywords:** veteran health care, mental health, suicide prevention, suicide intervention, suicide behavior

## Abstract

Despite the investment of public resources to fight staggering suicide rates among veterans, we know little about how veterans and service members in crisis communicate suicidal ideations, and what interventions they are willing to receive. We aim to identify communication and suicide intervention preferences of veterans and service members in times of crisis. Descriptive statistics were used to explore veterans communication of suicidal ideations. While 89.9% of participants indicated they were willing to speak to someone when having thoughts of suicide, less than 26% of participants indicated they were willing to bring up their thoughts with a crisis line or veterans organization. Rather, they indicate that family members (62.2%) and military friends (51.1%) would be their primary outreach. Logistic regression was used to determine whether or not preferred interventions varied by participant demographic characteristics. While the majority of participants indicated they were willing to allow intervention (88.6%), no one method was accepted by the majority of the population. The most accepted means of communication was to proactively contact a friend or family member about general life struggles (32.6%) or suicide-specific concerns (27.5%). Many participants were open to receiving resources (42.0%), suicide-specific mental health treatment (36.3%), and some sort of lethal means safety intervention (19.1%–26.4%). The age, marital status, and veterans status of participants significantly impacted what interventions they were willing to allow. We discuss the implications of these findings and the need for evidence-based, multimodal interventions in order to assist veterans in need.

## 1 Introduction

Suicide among military service members and veterans has been a primary concern of the mental health field, the Department of Defense (DoD), and the Department of Veterans Affairs (VA) for well over a decade (1–4). For the past 15 years, there have been extensive planning and recommendations directed to address the high rates of suicide. In 2019, the President’s Roadmap to Empower Veterans and End the National Tragedy of Suicide (PREVENTS) task force was formed by the White House and VA (5). In 2021, the White House also established a list of priority goals and activities for reducing military and veteran suicide (6). Yet, in the most recent National Veteran Suicide Prevention Annual Report, the rate of suicide among U.S. veterans was still 57.3% higher than the rate among non-veterans, and suicide was the second leading cause of death among veterans under the age of 45 (7).

We still know very little about who veterans talk to about their suicidality and what methods they use to communicate their struggles. This is important so that we might learn how to intervene earlier when a person initially expresses that they would be better off dead or they are thinking about killing themself. Of initial disclosures, over 89% of them were through relationships including friends, family, and domestic partners. The remaining 10% reported to medical professionals, mental health professionals and crisis hotlines (8). However, we know from previous veteran-centered work that the social and help seeking behaviors exhibited by military populations often differ from the general population due to the culture of military life (9–13).

The recommendations currently in place to decrease suicide among service members and veterans are evidence-based and actionable, but in order to maximize the effectiveness of these strategies, we need to understand how people are communicating that they are at increased risk of suicide and what is desired by those who are at increased risk of suicide. This study aims to identify those help seeking behaviors that are most commonly accepted by veterans when having thoughts of suicide. In addition, we explore how these help seeking behaviors differ by background characteristics and history of suicidal ideation. These insights can be used to inform future efforts in suicide prevention with veterans and service members, expanding our current understanding of the methods of communication and intervention efforts accepted by individuals. We will address this need by exploring the following research questions: Research questions:

1. How do veterans and service members communicate their thoughts of suicide?
2. What interventions are veterans and service members willing to allow when they have thoughts of suicide?
3. How do the allowable suicide prevention interventions differ by demographic characteristics?

## 2 Methods

This study was led by researchers at the non-profit organization Stop Soldier Suicide (SSS), and was done in partnership with Veteran Tickets Foundation (Vet Tix) (14), which offers veterans and service members free tickets to social events. Vet Tix has over 1.6 million users, or Vet Tixers. Within its online platform, the organization has a system for frequently asking short questionnaires to the Vet Tixers. SSS worked with Vet Tix to add four questions (Appendix 1) to its online survey platform related to suicide prevention. All research methods described in this study have been approved under an exempt status by the Advarra Institutional Review Board (Project approval number: 00065053).

### 2.1 Survey design

Survey questions were designed by SSS staff to help the organization better understand how to reach individuals with thoughts of suicide and what help they would be willing to receive. Veterans at SSS and Vet Tix reviewed and refined the proposed questions following discussions on question content and interpretation. Question interpretation and optimization discussions included staff at SSS, many of which belong to the target demographic. The final four questions administered were: (1) If you had thoughts of suicide, whom would you trust to talk with about those things, (2) If you had thoughts of suicide, how would you bring it up with others, (3) If you had thoughts of suicide, what would you allow others to do to help you, and (4) Have you ever had thoughts of killing yourself? At the end of the survey, participants were given the contact information for SSS in case they wished to use its 24/7 crisis and support services (see Appendix 1 for the full questionnaire). Vet Tix users that had previously completed surveys had preexisting demographic data available for use. Any participants who had not previously answered demographic questions were asked those prior to the four additional SSS questions.

### 2.2 Participant recruitment

The survey was made available to Vet Tix users for a 1 month period in the fall of 2022. A statement was included in the weekly email announcement notifying all Vet Tixer subscribers that the questions specific to SSS would be available. Additionally, when users logged into their account they were given the opportunity to click on a link to fill out Vet Tix questionnaires which included all Vet Tix questions and the four new SSS questions.

Vet Tixers who respond to the questionnaires are typically given one to five digital “appreciation coins” per question, which they can later use to have a greater chance of winning one of the more popular social event tickets on Vet Tix. For this study, Vet Tixers were given five appreciation coins per question and were permitted to answer or skip any combination of the four survey questions.

### 2.3 Data curation and cleaning

Vet Tix shared data in the form of a CSV file with Stop Soldier Suicide for all participants who consented to and responded to the SSS questionnaire. This data file included de-identified demographic information collected by Vet Tix, the responses to the SSS suicide prevention questions, and information on VA utilization based on another set of Vet Tix questions. The original data file included 99,262 participants. Additional data cleaning was performed to ensure that all data points were collected from those currently serving, veterans, or severely wounded veterans. Additionally, those individuals who reported being part of Law Enforcement Operations or the Space Force were excluded due to limited sample size and the increased probability of identifiable respondents. Individuals with an age greater than 100 or having reported serving more than 65 years were excluded due to concerns with data validity. This resulted in a sample size of 99,045. We then filtered the results to include individuals that responded to any of the four SSS questions, decreasing the sample size to 38,185. Lastly, the decision was made to filter the results to include only those individuals who responded to all 4 of the SSS suicide prevention questions, resulting in a final sample size of 31,180. Please note that the full SSS dataset (*N* = 38,195) was used in a follow-up analysis, and it was confirmed that the use of complete data (*N* = 31,180) did not change the narrative or substantially alter any of the descriptive statistics reported below.

### 2.4 Participant characteristics

Our sample includes members of the Army, Navy, Air Force, Marine Corps, and Coast Guard, but is largely composed of those currently or previously serving in the Army (44.6%) (Supplementary Table S1). Respondents were primarily veterans (79.9%) or severely wounded veterans (0.6%), although a substantial number were currently serving (19.5%).

Age was estimated based on years of service plus 18 for those who were enlisted and plus 22 for those who were ever officers. Estimated age ranged from 19 and 91 years (mean = 45.41, SD = 12.29 years) (Supplementary Table S1). A full description of participant demographics can be found in Supplementary Table S1.

### 2.5 Statistics analysis

All analyses were conducted in R (v4.2.2). Descriptive statistics were performed to determine whom veterans would feel comfortable reaching out to in crisis, how they would do so, and what interventions they would allow. In the survey (Appendix 1), participants were asked to select any responses that apply. As such, responses do not add to 100% in each case. To calculate these values, the number of participants that had selected each option were divided by the total number of participants (*N* = 31,180).

In order to evaluate whether or not particular demographic characteristics were likely to influence which interventions a participant may allow, logistic regression was performed. Each allowable intervention was evaluated as a response variable with covariates including: previous suicidal ideation, age, marital status, military status, and military branch.

## 3 Results

Of the 31,180 respondents, 18.7% said that they had prior thoughts of suicide, 67.1% said they did not have prior thoughts of suicide, and 14.1% preferred not to say (Supplementary Table S1).

### 3.1 Communicating suicidal ideation

**Figure 1:**
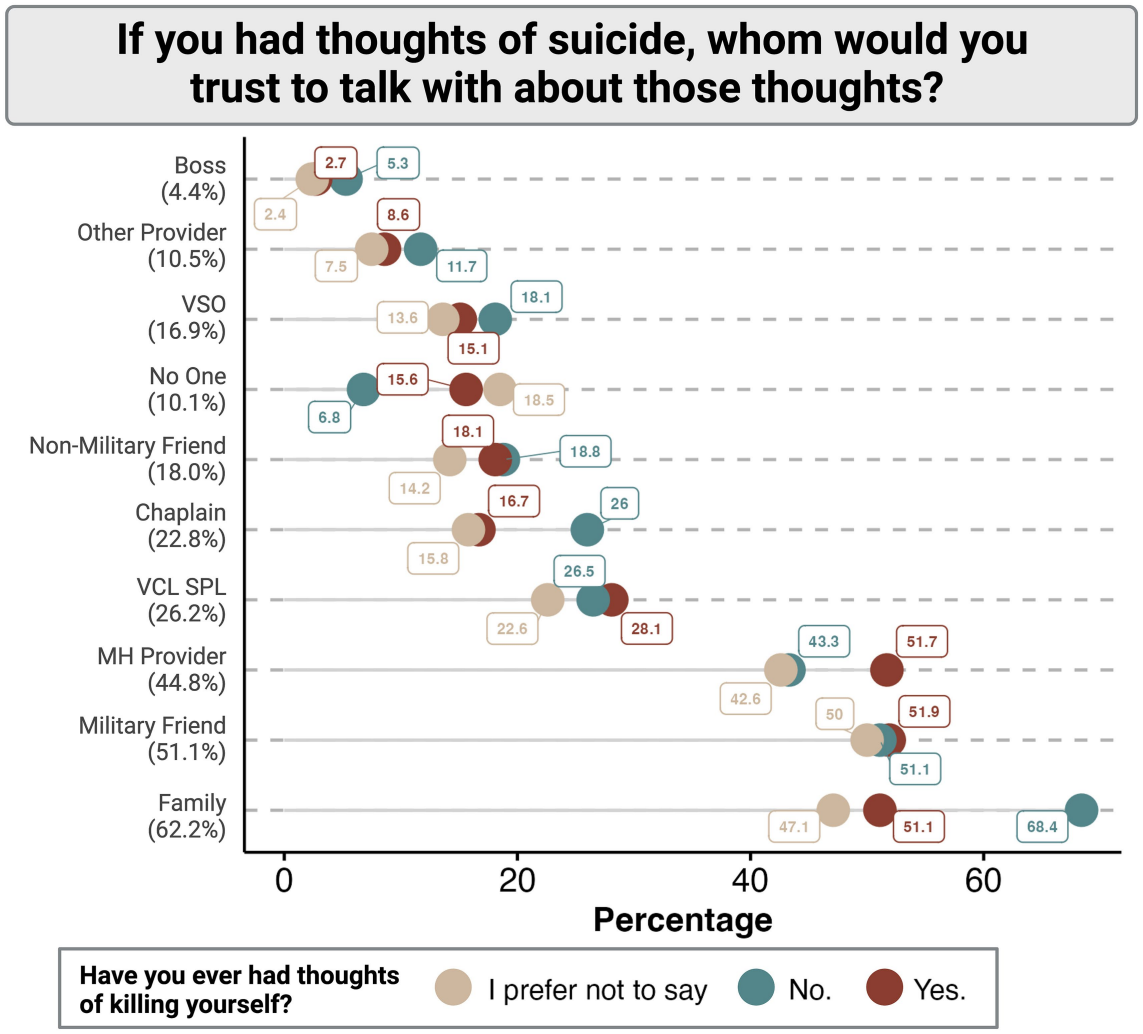
Distribution of trusted persons in communication of suicidal ideations among participants stratified by the presence or absence of previous suicidal thoughts. VSO, Veteran Serving Organization; VCL SPL, Veterans Crisis Line or National Suicide Prevention Line; MH, Mental health.

The majority of respondents stated that if they were to experience thoughts of suicide they would trust talking about those thoughts with family (62.2%) and military friends (51.1%), while a significant proportion also indicated that they would trust mental health providers with their thoughts about suicide (44.8%) (Figure 1). However, veterans and service members were far less likely to trust talking about their thoughts of suicide with non-military friends than military friends (18.0% vs. 51.1%). Respondents were also considerably less likely to talk about their thoughts of suicide with the Veterans Crisis Line (VCL) or National Suicide Prevention Line (SPL; 26.2%), Chaplains (22.8%), veteran service organizations (VSOs; 16.9%), other health care providers (10.5%), or their boss (4.4%). Only 10.1% of respondents stated that they would not trust talking with anyone about their thoughts of suicide.

When these findings were stratified by whether or not participants had experienced previous suicidal ideation (Figure 1), it was found that those individuals who did have a self-reported history of suicidal thoughts were equally as likely to reach out to family (51.1%), military friends (51.9%), or mental health providers (51.7%). Interestingly, those individuals who report no previous thoughts of suicide are more likely to report they would be willing to reach out to personal resources such as family, non-military friends, chaplains, or their boss than the respondents who did have previous thoughts of suicide (Figure 1).

**Figure 2:**
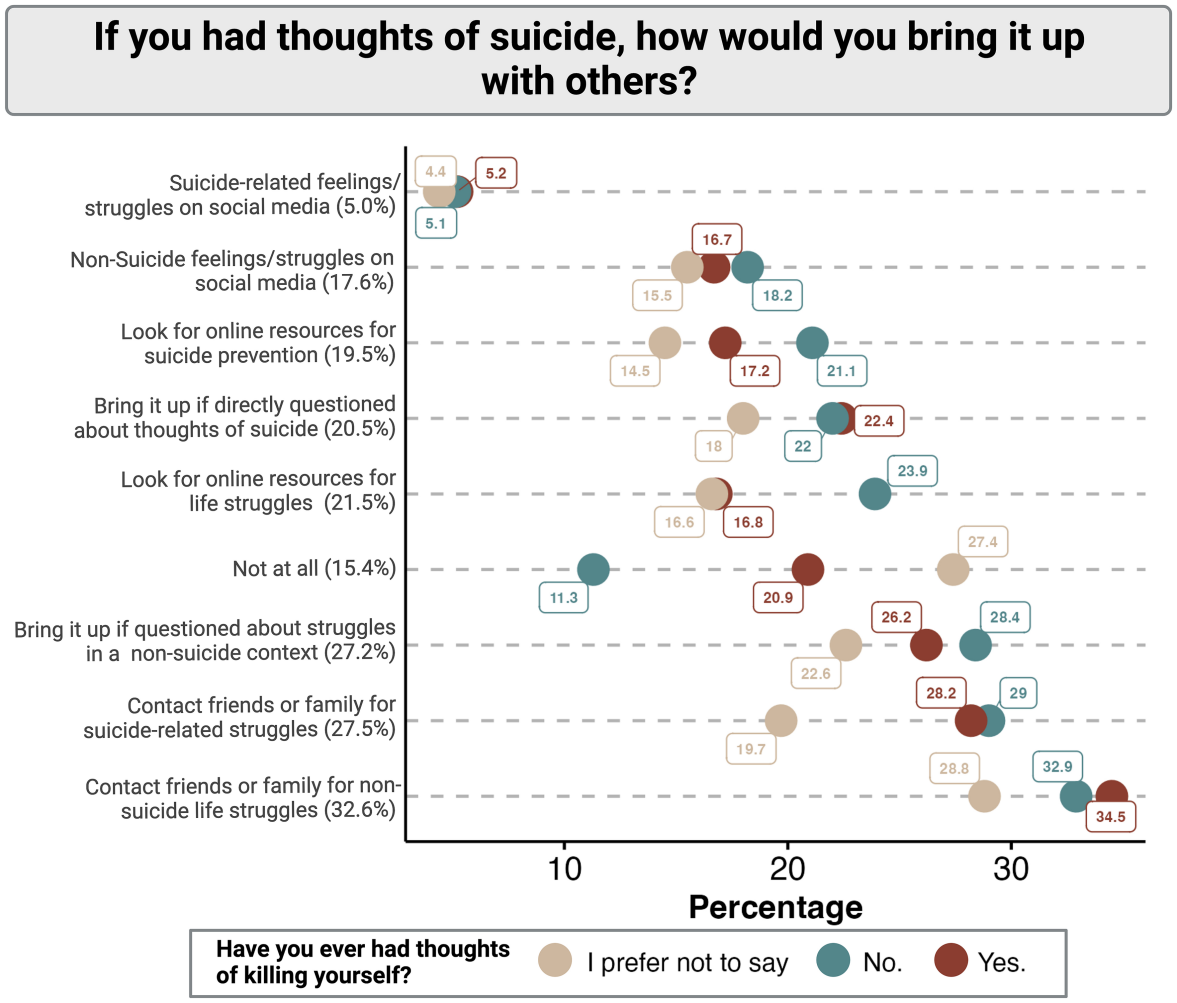
Distribution of suicide ideation communication methods among participants stratified by the presence or absence of previous suicidal thoughts.

If the veterans and service members hypothetically had thoughts of suicide, they said that they would most likely bring it up with others by proactively contacting a friend or family member and talking about their general life struggles (32.6%) or the suicide-specific thoughts they were experiencing (27.5%) (Figure 2). Another common theme was that when participants said they would reach out for help from friends and family, respond to questioning by others, or look online for resources, they said they would be more likely to address general life struggles than to directly address suicide (Figure 2). For example, while 17.6% of participants report a willingness to discuss their general life struggles on social media, that number decreased to 5% in reference to suicide-specific thoughts. Only 15.4% of participants stated that there were no methods by which they would bring up their struggles with others.

Generally, participants without a history of suicidal ideation report a lower willingness to reach out for help by all proposed means than those with a history of suicidal ideation (Figure 2). Those individuals that preferred not to confirm or deny a history of suicidal ideation were universally the least likely to report a willingness to communicate their struggles with others regardless of proposed source or method (Figures 1, 2).

### 3.2 Allowing for intervention

**Figure 3:**
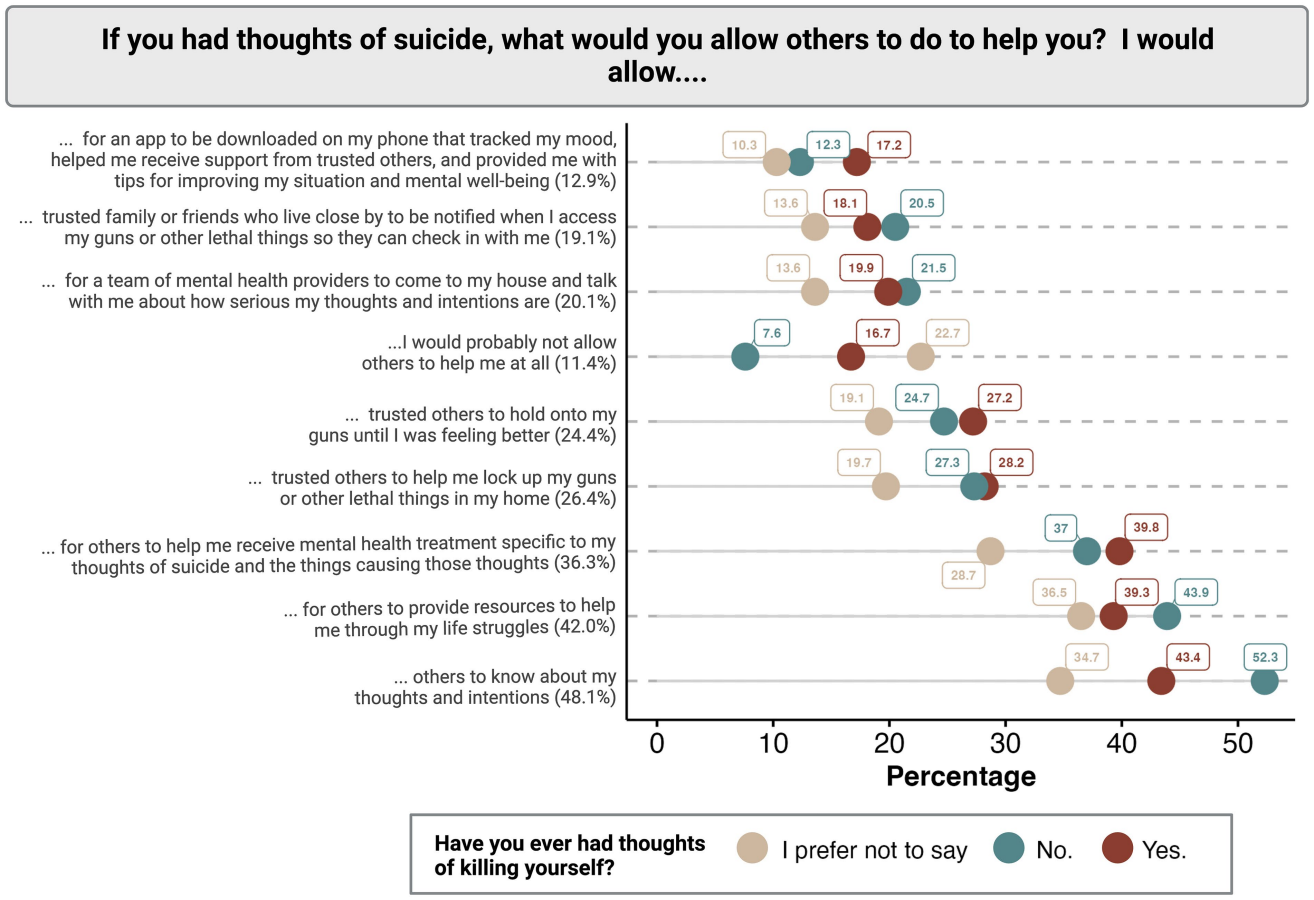
Distribution of accepted suicide interventions among participants stratified by the presence or absence of previous suicidal thoughts.

Nearly half of all respondents said they were willing to allow others to know about their thoughts and intentions of suicide (48.1%) or allow others to provide them with resources to help them through life struggles (42.0%) (Figure 3). While 36.3% of participants were willing to be supported in receiving mental health treatment specific to thoughts of suicide, only 20.1% of participants said they were willing to let mental health providers come to their home and discuss their thoughts and intentions. Only 12.9% of participants were willing to allow a downloaded app to track their mood and provide support and 11.4% were not willing to allow any type of intervention. Consistent with means of communicating suicidal ideation, individuals who prefer not to disclose their history of suicidal ideation were least likely to allow each proposed intervention.

A subset of questions specifically addressed gun safety, in which 26.4% of participants were willing to allow trusted others to help them lock up guns and other lethal things in their homes, 24.4% were willing to let trusted others hold their guns temporarily, and 19.1% were willing to allow trusted family or friends to be notified in the case that guns or other lethal means are accessed (Figure 3).

In our multivariate analysis (Table 1), we found various characteristics of veterans and service members that were significantly related to their willingness to allow different interventions when having thoughts of suicide. First of all, respondents who reported prior thoughts of suicide and those who preferred not to say whether they had prior thoughts of suicide generally said they would also be less willing to allow others to intervene if they were actively having thoughts of suicide compared to those who reported never having had prior thoughts of suicide. This is evident in both the large odds ratios (ORs) for the outcome of not allowing others to help (model 1), and the smaller than one ORs for all proposed interventions (models 2–9). There were a few exceptions to this trend. Compared to those with no prior thoughts of suicide, those who had prior thoughts of suicide had 1.41 times greater odds [95% confidence interval (CI): 1.30–1.53] of saying they would allow for an app to be downloaded on their phone that helps track their mood and receive support, 1.16 times greater odds (95% CI: 1.09–1.23) to receive suicide-specific mental health treatment, and 1.11 times greater odds (95% CI: 1.04–1.19) to allow trusted others to hold onto their guns until they felt better.

**Table 1:** Summary of logistic regression analysis.

		#1 …I would probably not allow others to help me at all	#2 … others to know about my thoughts and intentions	#3 … for others to provide resources to help me through my life struggles	#4 … for an app to be downloaded on my phone that tracked my mood, helped me receive support from trusted others, and provided me with tips for improving my situation and mental well-being	#5 … for a team of mental health providers to come to my house and talk with me about how serious my thoughts and intentions are	#6 … for others to help me receive mental health treatment specific to my thoughts of suicide and the things causing those thoughts	#7 … trusted others to help me lock up my guns or other lethal things in my home	#8 … trusted others to hold onto my guns until I was feeling better	#9 … trusted family or friends who live close by to be notified when I access my guns or other lethal things so they can check in with me
Prior suicide thoughts (Ref = “No”)	I prefer not to say	**3.560 (3.250–3.880)** ^***^	**0.480 (0.450–0.510)** ^***^	**0.760 (0.710–0.810)** ^***^	**0.790 (0.710–0.870)** ^***^	**0.580 (0.530–0.640)** ^***^	**0.710 (0.660–0.760)** ^***^	**0.630 (0.580–0.690)** ^***^	**0.700 (0.650–0.760)** ^***^	**0.590 (0.530–0.640)** ^***^
	Yes	**2.420 (2.220–2.650)** ^***^	**0.690 (0.650–0.730)** ^***^	**0.850 (0.800–0.900)** ^***^	**1.410 (1.300–1.530)** ^***^	**0.920 (0.850–0.990)** ^*^	**1.160 (1.090–1.230)** ^***^	1.020 (0.960–1.090)	**1.110 (1.040–1.190)** ^**^	**0.820 (0.760–0.890)** ^***^
Age (Ref = 65+)	18–24	**0.600 (0.460–0.780)** ^***^	**1.670 (1.430–1.960)** ^***^	**0.920 (0.780–1.070)** ^*^	**2.100 (1.660–2.680)** ^***^	**0.860 (0.710–1.040)** ^*^	**0.780 (0.660–0.910)** ^***^	**2.290 (1.920–2.720)** ^***^	**1.970 (1.640–2.360)** ^***^	**2.930 (2.400–3.580)** ^***^
	25–34	**0.760 (0.640–0.900)** ^**^	**1.620 (1.450–1.800)** ^***^	**0.820 (0.740–0.920)** ^***^	**2.020 (1.690–2.440)** ^***^	0.940 (0.830–1.080)	**0.770 (0.690–0.860)** ^***^	**1.980 (1.740–2.260)** ^***^	**1.830 (1.600–2.090)** ^***^	**2.440 (2.090–2.850)** ^***^
	35–44	**0.790 (0.670–0.920)** ^**^	**1.490 (1.350–1.650)** ^***^	**0.900 (0.820–1.000)** ^*^	**1.890 (1.590–2.260)** ^***^	0.930 (0.820–1.050)	**0.810 (0.740–0.900)** ^***^	**1.680 (1.490–1.900)** ^***^	**1.680 (1.480–1.910)** ^***^	**2.110 (1.820–2.450)** ^***^
	45–54	0.920 (0.790–1.070)	**1.250 (1.130–1.380)** ^***^	0.920 (0.840–1.020)	**1.530 (1.280–1.830)** ^***^	0.890 (0.790–1.000)	**0.800 (0.720–0.880)** ^***^	**1.290 (1.150–1.470)** ^***^	**1.370 (1.210–1.550)** ^***^	**1.560 (1.350–1.810)** ^***^
	55–64	0.860 (0.720–1.010)	**1.180 (1.060–1.320)** ^**^	1.050 (0.940–1.170)	**1.480 (1.230–1.780)** ^***^	0.980 (0.860–1.110)	0.950 (0.850–1.060)	**1.130 (0.990–1.280)** ^*^	**1.160 (1.010–1.330)** ^*^	**1.310 (1.120–1.530)** ^***^
Military status (Ref = “I am currently serving”)	Veteran	1.050 (0.940–1.170)	0.990 (0.930–1.050)	0.960 (0.900–1.030)	**0.910 (0.830–1.000)** ^**^	**0.870 (0.800–0.940)** ^***^	**0.920 (0.860–0.990)** ^***^	**0.850 (0.800–0.920)** ^***^	**0.850 (0.790–0.910)** ^***^	**0.850 (0.790–0.910)** ^***^
	Severely wounded veteran	1.240 (0.810–1.850)	**0.690 (0.510–0.940)** ^*^	0.960 (0.710–1.290)	0.770 (0.470–1.210)	0.710 (0.470–1.050)	0.860 (0.620–1.160)	**0.570 (0.380–0.820)** ^**^	0.790 (0.550–1.120)	0.690 (0.450–1.020)
Marital status (Ref = “Single”)	Cohabitation	**0.790 (0.640–0.960)** ^*^	1.020 (0.900–1.160)	0.940 (0.820–1.070)	1.150 (0.970–1.370)	0.990 (0.850–1.160)	0.970 (0.850–1.100)	**1.210 (1.050–1.400)** ^**^	**1.300 (1.130–1.510)** ^***^	1.100 (0.930–1.290)
	Divorced	0.930 (0.800–1.070)	1.000 (0.900–1.100)	0.970 (0.880–1.070)	1.030 (0.900–1.190)	1.030 (0.910–1.160)	0.950 (0.860–1.050)	1.100 (0.980–1.230)	**1.130 (1.010–1.270)** ^*^	1.050 (0.930–1.190)
	Married	**0.860 (0.770–0.970)** ^*^	1.040 (0.960–1.120)	**0.890 (0.830–0.970)** ^*^	**0.830 (0.750–0.930)** ^**^	**0.890 (0.810–0.980)** ^*^	**0.860 (0.800–0.930)** ^**^	**1.160 (1.060–1.260)** ^***^	**1.120 (1.030–1.230)** ^***^	**1.110 (1.010–1.220)** ^**^
	Separated	1.260 (0.960–1.620)	0.970 (0.800–1.180)	1.020 (0.840–1.240)	1.000 (0.760–1.310)	1.000 (0.780–1.270)	0.910 (0.740–1.110)	**1.270 (1.020–1.570)** ^*^	**1.350 (1.090–1.680)** ^**^	1.140 (0.890–1.450)
	Widowed	0.980 (0.710–1.350)	0.830 (0.670–1.030)	0.980 (0.790–1.220)	1.120 (0.810–1.520)	0.990 (0.760–1.280)	0.880 (0.700–1.090)	0.850 (0.640–1.110)	1.080 (0.830–1.400)	0.900 (0.650–1.220)
Military Branch (Ref = “Marine Corps”)	Air Force	**0.830 (0.730–0.940)** ^**^	1.040 (0.960–1.130)	**1.100 (1.010–1.200)** ^**^	**1.130 (1.000–1.280)** ^**^	**1.140 (1.030–1.270)** ^**^	**1.140 (1.050–1.250)** ^***^	1.020 (0.930–1.120)	1.070 (0.970–1.170)	1.080 (0.970–1.210)
	Army	**0.900 (0.810–1.010)** ^*^	1.050 (0.970–1.130)	0.950 (0.880–1.020)	**1.100 (0.990–1.240)** ^*^	**1.150 (1.040–1.260)** ^**^	**1.090 (1.010–1.180)** ^**^	1.010 (0.930–1.100)	1.080 (0.990–1.180)	**1.110 (1.010–1.220)** ^*^
	Coast Guard	0.850 (0.630–1.130)	1.150 (0.960–1.370)	**1.190 (1–1.420)** ^*^	**1.290 (1.010–1.650)** ^*^	**1.280 (1.040–1.580)** ^*^	**1.180 (0.990–1.410)** ^*^	1.060 (0.880–1.290)	0.900 (0.730–1.100)	1.140 (0.920–1.410)
	Navy	0.890 (0.790–1.010)	**1.090 (1.000–1.180)** ^*^	1.040 (0.960–1.130)	**1.120 (0.990–1.270)** ^*^	**1.180 (1.060–1.310)** ^***^	**1.160 (1.070–1.270)** ^***^	0.970 (0.880–1.070)	1.030 (0.930–1.130)	1.100 (0.990–1.220)
Education (Ref = “High school or equivalent”)	Trade, technical, or vocational school	0.920 (0.770–1.100)	**0.880 (0.780–0.990)** ^*^	**1.270 (1.130–1.430)** ^***^	1.130 (0.940–1.370)	0.940 (0.800–1.100)	1.090 (0.960–1.230)	1.050 (0.920–1.200)	1.030 (0.900–1.190)	0.990 (0.850–1.160)
	Some college credit, no degree	0.900 (0.780–1.030)	1.020 (0.930–1.110)	**1.150 (1.050–1.260)** ^**^	**1.180 (1.030–1.370)** ^*^	1.090 (0.970–1.220)	**1.130 (1.030–1.250)** ^*^	1.040 (0.940–1.150)	1.050 (0.950–1.170)	1.040 (0.930–1.170)
	Associate degree	**0.850 (0.730–0.990)** ^*^	1.040 (0.940–1.140)	**1.210 (1.100–1.340)** ^***^	1.150 (0.990–1.350)	1.090 (0.960–1.230)	**1.190 (1.070–1.320)** ^***^	1.030 (0.920–1.150)	0.960 (0.860–1.080)	1.020 (0.900–1.160)
	Bachelor’s degree	**0.840 (0.740–0.970)** ^*^	1.070 (0.980–1.170)	**1.280 (1.170–1.410)** ^***^	**1.310 (1.140–1.510)** ^***^	**1.230 (1.090–1.370)** ^***^	**1.240 (1.130–1.360)** ^***^	1.020 (0.920–1.130)	0.970 (0.880–1.080)	1.010 (0.900–1.130)
	Master’s degree	**0.770 (0.660–0.890)** ^***^	**1.130 (1.020–1.240)** ^*^	**1.330 (1.210–1.470)** ^***^	**1.500 (1.300–1.750)** ^***^	**1.280 (1.130–1.450)** ^***^	**1.390 (1.250–1.540)** ^***^	1.100 (0.990–1.230)	1.010 (0.910–1.140)	1.020 (0.900–1.160)
	Doctorate degree	0.820 (0.600–1.090)	**1.210 (1.010–1.450)** ^*^	**1.520 (1.270–1.830)** ^***^	**1.480 (1.130–1.920)** ^**^	**1.430 (1.150–1.770)** ^***^	**1.280 (1.060–1.540)** ^*^	**1.230 (1.010–1.500)** ^*^	0.970 (0.790–1.200)	1.100 (0.870–1.370)
	Professional degree	**0.580 (0.380–0.850)** ^**^	1.190 (0.950–1.490)	**1.470 (1.170–1.840)** ^***^	1.320 (0.930–1.830)	1.080 (0.810–1.430)	**1.510 (1.200–1.900)** ^***^	1.060 (0.820–1.370)	0.970 (0.740–1.260)	0.860 (0.630–1.150)
Income (Ref = “Less than $25,000”)	$25,000–$49,999	1.030 (0.870–1.220)	1.020 (0.910–1.150)	0.990 (0.880–1.120)	1.060 (0.880–1.270)	1.010 (0.870–1.170)	0.990 (0.870–1.120)	0.940 (0.820–1.090)	**1.180 (1.020–1.370)** ^*^	1.000 (0.860–1.170)
	$50,000–$74,999	0.910 (0.770–1.090)	1.070 (0.950–1.200)	1.050 (0.940–1.190)	1.110 (0.930–1.320)	1.040 (0.890–1.200)	1.040 (0.920–1.170)	1.110 (0.970–1.270)	**1.260 (1.090–1.460)** ^**^	1.030 (0.890–1.210)
	$75,000–$99,999	0.930 (0.780–1.110)	1.090 (0.970–1.240)	1.090 (0.960–1.230)	1.100 (0.920–1.320)	1.070 (0.920–1.250)	1.050 (0.930–1.190)	1.110 (0.970–1.280)	**1.330 (1.150–1.540)** ^***^	1.070 (0.910–1.250)
	$100,000–$149,999	0.970 (0.820–1.170)	**1.140 (1.010–1.290)** ^*^	0.990 (0.870–1.120)	1.080 (0.900–1.300)	1.030 (0.890–1.210)	1.030 (0.910–1.170)	1.100 (0.960–1.270)	**1.350 (1.160–1.570)** ^***^	1.030 (0.880–1.210)
	$150,000–$199,999	0.960 (0.770–1.190)	1.130 (0.980–1.300)	1.060 (0.920–1.230)	1.060 (0.860–1.310)	1.130 (0.950–1.350)	1.040 (0.900–1.210)	**1.210 (1.030–1.420)** ^*^	**1.440 (1.210–1.700)** ^***^	**1.260 (1.050–1.510)** ^*^
	$200,000+	0.960 (0.750–1.230)	**1.380 (1.170–1.610)** ^***^	0.950 (0.810–1.110)	1.030 (0.810–1.300)	1.130 (0.930–1.380)	1.040 (0.890–1.230)	1.130 (0.940–1.360)	**1.360 (1.120–1.640)** ^**^	1.130 (0.920–1.390)

Results are reported as odds ratios with 95% confidence intervals. Statistical significance is indicated with bold face text, and levels of significance is indicated with an asterisk (^*^p < 0.05, ^**^p < 0.01, and ^***^p < 0.001).

The types of allowable interventions varied by age (Table 1). Younger age groups (18–44 years) had significantly greater odds of saying they would allow others to know about their thoughts and intentions compared to older age groups (model 2). Younger age groups were also more willing to download an app to help them improve their mental health; and they had greater odds for saying they would allow for their guns to be locked up, allow trusted others to hold onto their guns, or allow trusted others to be notified when accessing their guns (models 7–9). However, age groups ranging from 18–54 had significantly lower odds than those 65 and older to say they were willing to receive suicide-specific mental health treatment. Younger age groups from ages 18–44 also had significantly lower odds than those 65 and older to be willing to allow others to provide them with resources to help with life struggles.

For many of the suicide prevention interventions, veterans had lower odds of allowing them compared to those who are currently serving in the military (Table 1). This was specifically the case for allowing for a mental health app to be downloaded on their phone (OR: 0.91; 95% CI: 0.83–1.00), allowing a team of mental health providers come to their house (OR: 0.87; 95% CI:0.80–0.94), receiving mental health treatment specific to suicide (OR: 0.92, 95% CI: 0.86–0.99), and all three interventions that were presented related to guns.

Marital status was also significantly related to the odds of being willing to allow for different interventions (Table 1). Those who were married had significantly lower odds than those who were single to allow others to provide resources, a mental health app to be downloaded on their phone, or for receiving a visit from a mental health team or suicide-specific mental health treatment. However, those who were married had significantly higher odds than those who were single to allow trusted others to help lock up guns or other lethal means, hold onto their guns, or be notified when accessing guns or other lethal means.

As education level increases, general patterns can be seen indicating an increased likelihood to accept resources, download mobile apps to track emotional wellbeing, and allow for in home and out of home mental health care (Table 1). However, education level was not consistently related to the likelihood of participants to allow firearm related interventions (i.e., trusted others to help lock up firearms, trusted others to hold onto guns, or trusted others to receive firearm access notifications). Interestingly, income level was only a significant predictor at all levels for the intervention of allowing trusted others to hold onto firearms, which increased with income (Table 1).

## 4 Discussion

The results from this study align with and add new insights to the existing literature. A previous study in the non-military population reported that a majority of participants with a history of suicidal ideation informally disclosed their thoughts with friends (73.3%), family (58.8%) or domestic partners (75.6%). Formal disclosures occurred with medical professionals (35.9%), mental health professionals (48.1%) or crisis hotlines (16.0%) (8). Our study indicates that service members and veterans may differ slightly from one another in the trusted individuals with whom they communicate suicidal thoughts. Our results closely match those based on the general population and a previous study on veterans with diagnosed psychological problems in terms of family, mental health professionals and crisis hotlines (8, 12). However, while the general public is willing to trust their friends with their thoughts of suicide at a high rate, service members and veterans are far more likely to trust their military friends (51.1%) than their non-military friends (18.0%). Additionally, they are less likely to trust general medical providers with their thoughts of suicide (10.5% vs. 35.9% in the general public). Participants in the study by Ammerman et al. (8) also reported that there was no significant difference in the level of perceived helpfulness of recipient reaction between disclosures in formal and informal settings. Even though we did not ask about this in our study, this implies that any attempts to communicate suicidal thoughts, whether in a formal or informal setting, could be equally as productive and should be considered suicide prevention resources.

There are striking differences in the types of interventions allowed by those who report they have, or have not had previous suicidal thoughts. Participants who report not having previous suicidal thoughts indicate that they would allow trusted others to know about their thoughts, offer resources, receive gun notifications, or allow provider to enter their home. Alternatively, those participants who report previous suicidal thoughts are more likely to allow mental health treatment, for an app to track their mood and offer resources, or a trusted individual to hold or temporarily lock up their guns. These results indicate that participants who have had the experience of previously requiring suicide-based intervention may be in favor of action-based interventions that are not particularly invasive to their privacy which may require others to come to their home or receive unwanted notifications about their habits.

The subset of the miliary population that does not wish to disclose the presence or absence of prior suicidal ideations has been shown to be at particularly high risk. Our data suggests that these service members are also less likely to communicate current suicidal thoughts or allow any type of intervention on their behalf. This aligns with the outcomes form The National Health and Resilience in Veterans study, which reported that individuals who declined to respond to a history of self-injurious thoughts and behaviors were more likely to screen positive for PTSD, increased trauma burden, loneliness, and other risk factors associated with suicide (15). Additionally, a previous study utilizing the PHQ-9 indicated that 71.6% of suicides were among patients who responded “not at all” when asked if they had “thoughts that you would be better off dead, or thoughts of hurting yourself in some way” (16). These data together display the importance in providing care to those individuals who are not willing to disclose their history with suicidal ideation.

While a great deal of VA and DOD efforts toward suicide prevention have gone into the effective diagnosis and treatment of mental health issues, there has more recently been an acknowledgement of the importance of lethal means safety (17, 18). Nearly 70% of all service member suicides and 50% of military family member suicides used a firearm (19–21). However, veterans have reported that holding lethal means safety discussions in a health setting with high-risk individuals is acceptable when done properly (22), indicating that veterans and service members are open to potential gun-based interventions. As nearly 25% of respondents in our study indicate they would allow one or more interventions including having a trusted individual lock up their guns, hold their guns temporarily, or receive notifications in the event of a lethal-means access event, the potential for crisis response involving safer storage of lethal-means is a promising avenue for future suicide prevention research.

While respondents above the age of 55 were significantly more likely to allow mental health treatment than those under 55, there were a number of alternative interventions that were more commonly accepted by the younger respondents. With a wave of veterans at increased suicide risk coming from our more recent operations, specifically Operation Enduring Freedom, Operation Iraqi Freedom and Operation New Dawn (19), the mental health field must adapt the available suicide prevention resources to be inclusive of this more recent generation in order to decrease suicide rates in the coming decades. With decreasing age, respondents were more likely to allow lethal-means safety interventions, the use of technology-based services, and other less direct mental health treatments. Future suicide prevention research targeting veterans of this era should focus on “new-age” technologies as well and how we can adapt our current practices to meet the needs our veterans that are not currently being met.

### 4.1 Limitations

This study includes a subset of veterans and active duty members that have self-selected to participate in Vet Tix programs. The survey was administered online through the Vet Tix webpage, which requires internet access and targets the population of veterans that use technology comfortably and therefore may not be generalizable to the greater veteran population. Due to limitations in the survey platform, participants were able to co-select responses equivalent to “none” and additional responses indicating acceptance. For example, a participant could indicate that they were not likely to accept help from anyone, but also select “military friends” as a second option. While we feel this information is insightful as two potential avenues of action, it is also possible that one some occasions, the multiples selections were an error due to the design of the survey.

While self-report data is common in suicidality research, it is worth noting that covariate data collection took place prior to survey administration, and may therefore be influenced by time disparities between demographic reports and self-reported behaviors. Additionally, we were unable to collect data on psychiatric history, such as psychiatric diagnoses, treatments, and other factors that may influence an individual’s help seeking and intervention behaviors.

## 5 Conclusion

This study underscores the need for basic mental health awareness training in the broader military, veteran, and general populations, so that there is a clear understanding of what to do next if a loved one shares thoughts of suicide or significant life struggles. In addition, although some assume that a veteran would never be willing to part with their weapon, these results suggest that some service members and veterans would be open to lethal means safety, especially at times of risk. Finally, given that some individuals are more open to traditional mental health treatment while others are more open to technological and app-based resources, it is important that the field continue to pursue evidence-based and multimodal methods of intervening, so that resources can be made available that are desirable or a good fit for the individual veteran or service member.

## Data availability statement

The datasets presented in this study can be found in online repositories. The names of the repository/repositories and accession number(s) can be found at: https://github.com/Stop-Soldier-Suicide/Vet-Communication-Intervention.git.

## Ethics statement

The studies involving humans were approved by Advarra Institutional Review Board (Project approval number: 00065053). The studies were conducted in accordance with the local legislation and institutional requirements. The participants provided their written informed consent to participate in this study.

## Author contributions

JR, KeH, and SVB contributed to conception and design of the study. SW, JR, and KaH organized and executed data collection. JR and AB organized the data. AB performed the statistical analysis. AB and JR wrote the first draft of the manuscript. All authors contributed to the article and approved the submitted version.
