# Supplementary material for: Suicide risk communication and intervention preferences for veterans and service members
Source: Front Public Health. 2023 Nov 21;11:1215925. doi: 10.3389/fpubh.2023.1215925 (PMC10703287; doi:10.3389/fpubh.2023.1215925)
Supplement: Supplementary file 1 [file Data_Sheet_1.docx]

Supplementary Material

**Suicide risk communication and intervention preferences for veterans and service members.**

# Appendix 1: Stop Soldier Suicide Survey Administered through Vet Tix

1. This is a special survey for Suicide Prevention Awareness Month.
    If you had thoughts of suicide, whom would you trust to talk with about those thoughts? (Think of specific people, and *check all that apply*)

Family member

Friend who has served in the military

Friend who has not served in the military

Commander or boss

Chaplain or religious leader

Mental health provider

Other health provider

[Veterans Crisis Line](https://www.veteranscrisisline.net/) or [National Suicide Prevention Lifeline](https://suicidepreventionlifeline.org)

Veterans service organization (e.g., [Stop Soldier Suicide](https://stopsoldiersuicide.org/))

Probably no one

2. If you had thoughts of suicide, how would you bring it up with others? (*Check all that apply*)

I would mention some of my general feelings and life struggles on social media, but not mention thoughts of suicide specifically.

I would mention my thoughts of suicide or being better off dead on social media.

I would look online for resources to get help with my life struggles.

I would look online for resources specific to suicide prevention.

I would text, email, or call a friend or family member and share my general feelings and life struggles, but probably not my thoughts of suicide.

I would text, email, or call a friend or family member and share that I was thinking about suicide.

I would bring it up with others I trusted if they asked how I was doing in general.

I would bring it up with others I trusted if they directly asked whether I was having thoughts of suicide.

I would probably not bring it up with others at all.

3. If you had thoughts of suicide, what would you allow others to do to help you? (*Check all that apply*)

I would allow others to know about my thoughts and intentions.

I would allow trusted others to help me lock up my guns or other lethal things in my home.

I would allow trusted family or friends who live close by to be notified when I access my guns or other lethal things so they can check in with me.

I would allow trusted others to hold onto my guns until I was feeling better.

I would allow for a team of mental health providers to come to my house and talk with me about how serious my thoughts and intentions are.

I would allow for an app to be downloaded on my phone that tracked my mood, helped me receive support from trusted others, and provided me with tips for improving my situation and mental well-being.

I would allow for others to help me receive mental health treatment specific to my thoughts of suicide and the things causing those thoughts.

I would allow for others to provide resources to help me through my life struggles.

I would probably not allow others to help me at all.

4. Have you ever had thoughts of killing yourself? (Your responses are confidential. If you are currently having thoughts of suicide you can get free, immediate, and confidential help 24/7 from [Stop Soldier Suicide](https://stopsoldiersuicide.org/?utm_source=program&utm_medium=partner&utm_campaign=partner+referral&utm_content=vettix) (866-481-5223).)

Yes

No

Skip, I prefer not to say.

# Supplemental Table 1: Demographics of survey participants following data curation.

| **Measure** | **Categories** | **Sample Size (N=31,180)** | **Percentage** | **Measure** | **Categories** | **Sample Size (N=31,180)** | **Percentage** |
| --- | --- | --- | --- | --- | --- | --- | --- |
| **Estimated Age^1^** | 18-24 | n = 1,239 | 4.0% | **Branch** | Air Force | n = 6,544 | 21% |
|  | 25-34 | n = 5,580 | 17.9% |  | Army | n = 13,901 | 44.6% |
|  | 35-44 | n = 8,892 | 28.5% |  | Coast Guard | n = 602 | 1.9% |
|  | 45-54 | n = 8,764 | 28.1% |  | Marine Corps | n = 3,757 | 12% |
|  | 55-64 | n = 4,597 | 14.7% |  | Navy | n = 6,376 | 20.4% |
|  | 65+ | n = 2,102 | 6.8% | **Suicidal Thoughts** | Yes | n = 5,846 | 18.7% |
| **Status** | Severely Wounded Veteran | n = 190 | 0.6% |  | No | n = 20,936 | 67.1% |
|  | Veteran | n = 24,904 | 79.9% |  | Prefer not to say | n = 4,398 | 14.1% |
|  | Currently Serving | n = 6,086 | 19.5% | **Education Level** | High School or equivalent | n = 2,730 | 8.8% |
| **Household Income** | Less than $25K | n = 1,410 | 4.5% |  | Trade/technical/vocational training | n = 1,962 | 6.3% |
|  | $25,000-$49,999 | n = 5,368 | 17.2% |  | Some college credit/ no degree | n = 6,773 | 21.7% |
|  | $50,000-$74,999 | n = 7,885 | 25.3% |  | Associate Degree | n = 4,564 | 14.6% |
|  | $75,000-$99,999 | n = 6,230 | 20% |  | Bachelor’s Degree | n = 8,621 | 27.6% |
|  | $100,000-$149,999 | n = 6,663 | 21.4% |  | Master’s Degree | n = 5,557 | 17.8% |
|  | $150,000-$199,999 | n = 2,255 | 7.2% |  | Doctorate Degree | n = 613 | 2% |
|  | $200,000 + | n = 1,369 | 4.4% |  | Professional Degree | n = 360 | 1.2% |
| **Marital Status** | Single | n = 3,447 | 11.1% | **Years Served** | 0-2 | n = 2,403 | 7.7% |
|  | Separated | n = 477 | 1.5% |  | 3-5 | n = 8,996 | 28.9% |
|  | Divorced | n = 3,496 | 11.2% |  | 6-10 | n = 7,547 | 24.2% |
|  | Cohabitation | n = 1,342 | 4.3% |  | 11-20 | n = 6,176 | 19.8% |
|  | Married | n = 22,023 | 70.6% |  | 21-40 | n = 5,972 | 19.2% |
|  | Widowed | n = 395 | 1.3% |  | 41-60 | n = 86 | 0.3% |
| **Highest Rank** | Non-officer | n = 27,302 | 87.6% | ^1^Age is an estimate based on 18+years of service for the enlisted and 22+years of service for those whose highest rank was an officer. | | | |
|  | Warrant Officer | n = 431 | 1.4% |  |  |  |  |
|  | Commissioned Officer | n = 3,447 | 11% |  |  |  |  |
